# EMT and tumor metastasis

**DOI:** 10.1186/s40169-015-0048-3

**Published:** 2015-02-26

**Authors:** Sarah Heerboth, Genevieve Housman, Meghan Leary, Mckenna Longacre, Shannon Byler, Karolina Lapinska, Amber Willbanks, Sibaji Sarkar

**Affiliations:** Cancer Center, Department of Medicine, Boston University School of Medicine, Boston, MA USA; School of Human Evolution and Social Change, Arizona State University, Tempe, AZ USA; Harvard Medical School, Boston, MA USA

## Abstract

EMT and MET comprise the processes by which cells transit between epithelial and mesenchymal states, and they play integral roles in both normal development and cancer metastasis. This article reviews these processes and the molecular pathways that contribute to them. First, we compare embryogenesis and development with cancer metastasis. We then discuss the signaling pathways and the differential expression and down-regulation of receptors in both tumor cells and stromal cells, which play a role in EMT and metastasis. We further delve into the clinical implications of EMT and MET in several types of tumors, and lastly, we discuss the role of epigenetic events that regulate EMT/MET processes. We hypothesize that reversible epigenetic events regulate both EMT and MET, and thus, also regulate the development of different types of metastatic cancers.

## Review

### EMT and MET: an introduction

The epithelial-mesenchymal transition (EMT) was originally described in the context of normal cell differentiation during early development [1]. Evolutionarily, the development of increased differentiation of mesenchymal cells allowed for the organization of highly specialized tissues and organ systems in various organisms. As such, it is not surprising that the molecular pathways classically associated with EMT, including Snail/Slug, Twist, Six1, Cripto, TGF-β, and Wnt/β-catenin, are highly conserved across species [1]. More recently, the role of adherent EMT in pathogenesis of fibrosis and metastasis of certain carcinogenic tumors has been described [1-13]. This new paradigm has challenged the field to more explicitly define EMT. Doing so may help researchers more accurately assess the relationship between the normal process of cell differentiation and the analogous pathological EMT processes. Such EMT processes occur in both epithelial and non-epithelial cancer, and while the mechanistic distinction of EMT in these cell types is worthy of further consideration, it is beyond the scope of this work. Here, we adopt a broad definition of EMT that includes molecular changes, decreased cell-cell recognition and adhesion, and increased potential for cell motility.

Embryonic development is a process that involves growth and differentiation. A significant portion of this process involves cellular differentiation and tissue formation, and once all major structures are formed, growth and weight gain take over. The process of a single cell either differentiating into increasingly specialized cells or growing and dividing into identical cells is programmed into its underlying epigenetic controls [14]. The particular constellation of regulatory changes that enable EMT drive a normal process of increased differentiation in developing populations of cells within an organism. However, when similar epigenetic modifications occur in cancer cells, these cells become metastatic.

It is important to note that before these cancer cells are able to metastasize, they must first overcome anoikis, a form of programed cell death initiated when anchorage-dependent cells (integrins) detach from the surrounding ECM [15]. Under normal conditions, when integrins on the epithelial cell surface come in contact with the ECM, FAK is activated by phosphorylation, which in turn triggers a phosphorylation cascade ending with the activation of Akt, thus promoting cell survival. If the integrin should lose contact with the ECM, the cell survival signals cease, leaving pro-apoptotic proteins such as Bad uninhibited and able to initiate cell death. Cancer cells can overcome anoikis in a variety of ways that are often related to EMT. For example, a loss of E-cadherin expression and an increase in N-cadherin expression is correlated with anoikis resistance and increased invasiveness [16]. It has also been shown that disregulation of growth factor receptors can lead to anoikis resistance.

To summarize, in order to migrate, cancer cells must activate genes necessary for differentiation, slow down proliferation events, activate anti-apoptotic mechanisms as initiating differentiation can induce some apoptotic pathways, alter cellular characteristics from epithelial to mesenchymal, down-regulate the receptors that aid in cell-to-cell attachment, up-regulate the cell adhesion molecules that help in cell movement, degrade cell-to-cell junctions, and activate proteases at the cell surface in order to cut through the extracellular matrix. Different populations of cancer cells possess varying epigenetic patterns that promote these changes, and each pattern holds different clinical significance. The complexity of EMT and metastasis lies in the heterogeneity of the population: not all cells will undergo EMT simultaneously, and not all cells that have undergone EMT will successfully metastasize. Cancer progenitor cell characteristics, environmental factors, extracellular and intracellular signaling, and epigenetic changes all influence whether a cell undergoes EMT and metastasis.

Two hypotheses currently attempt to explain EMT and metastasis [17]. In the first hypothesis, cancer progenitor cells present in a tumor do not undergo EMT simultaneously, so the cancerous population contains cells at different stages of differentiation. However, these stages are not fixed. Cancer progenitor cells at any given stage of differentiation can undergo EMT to achieve a further stage of differentiation and develop into an advanced grade of cancer. Essentially, although these grades are different, they arise from the same progenitor cell and undergo differential EMT at different time points. The second hypothesis suggests that some cancer progenitor cells initially undergo EMT and then metastasize following clonal expansion. In this instance, a metastatic tumor will share a signature with the cell that originally underwent EMT, and thus, every cancer grade should come from a different progenitor cell. Recent studies in breast cancer have observed that heterogenic metastatic breast cancer tumors are derived from a few cancer progenitor cells also support the first hypothesis [18,19]. However, it is still possible for the heterogeneity of metastatic cancer to be generated by many cancer progenitor cells differentiating at different times to produce different cancer grades [20]. The contribution of only a few cancer progenitor cells to metastatic breast cancer is inconsistent with findings that metastatic breast cancer cells have high genetic diversity [21]. This anomaly was recently addressed when it was demonstrated that heterogeneity in cancer does not evolve from random genetic changes but rather is orchestrated by an evolutionary conserved and organized mechanism [22]. This organized mechanism involves the distinct pattern of epigenetic changes known as EMT [17,18,23].

Another important precondition for successful metastasis is the mesenchymal-epithelial transition (MET). Cancer cells that have undergone EMT and traveled to other parts of the body must have a mechanism that allows them to infiltrate other tissues and produce new, clinically significant tumor sites. To do this, they must first regain epithelial characteristics so as to anchor themselves in the surrounding tissue. An example of this phenomenon is observed in induced pluripotent stem cells (iPSCs). Recent studies show that producing iPSCs by increasing the expression of factors that induce MET also suppresses EMT mediators [24]. This elegant modulation between metastatic and successful implantation in distant tissues further supports the highly organized, evolved EMT/MET hypothesis of metastasis.

## Mechanisms of EMT

### Signaling

Several transcription factors are up-regulated in metastatic cells that are undergoing EMT, including Snail, Twist, Zeb, and others. TGF-β plays a large role in activating Snail, which in turn down-regulates cadherin-16 and HNF-1β, and this process is involved in the epithelial to mesenchymal transition [25]. Because TGF-β also induces apoptosis, cancer cells must protect themselves from this cell-death pathway. Interestingly, in addition to inducing EMT, Snail up-regulates Akt and Bcl-xL, which inhibit TGF-β-induced apoptosis in cancer cells [26]. However, in conferring resistance to apoptosis, Snail has been shown to inhibit cell cycle progression through the down-regulation of Cyclin D2. When the cancer cells are going through this differentiation, a reduction in their cell cycle progression is expected [27]. In a tumor microenvironment, Snail can be activated through multiple pathways, including HIF1, HIF2, and Notch in response to hypoxia as well as NF-κB and TGF-β in response to inflammation [28,29]. IGFR has been shown to induce EMT through NF-κB and Snail in mammary epithelial cells. IGFR also up-regulates Zeb in prostate carcinoma and can activate latent TGF-β1 to induce EMT [30,31]. It has been proposed that CCN6 (WISP3) can suppress EMT in breast cancer cells by inhibiting Zeb1 through the modulation of IGF-1 signaling [32]. During EMT, TGF-β can also induce expression of Cox-2, a gene frequently up-regulated in breast cancer. Elevated expression levels of Cox-2 have been associated with increased prostaglandin E_2_ production, and Cox-2 is believed to be an antagonist of Smad2/Smad3 [33]. In particular, expression levels of HIF-1α, a protein that plays a central role in the development of aggressive, mesenchymal phenotypes in hypoxic and inflammatory environments, have been shown to induce IL-8, VEGF, and Twist1 expression, and thus EMT [34]. ERβ1 has been shown to repress EMT by interfering with HIF-1α-mediated transcription of VEGF-A through the ERE and HRE response elements in the VEGF-A promoter; thus, low levels of ERβ1 result in an EMT. VEGF-A is thought to be involved in EMT by promoting nuclear localization of Snail1 [35].

There are also several ways alternative splicing can play a role in EMT regulation. This was first established in a study of pancreatic cancer that found specific splice variants of CD44 in metastasized cancer cells that were not present in the primary tumor cells [36]. It was later found that splicing factor ESRP1 in epithelial cells acts to inhibit the CD44 isoform switching from an epithelial variant to the mesenchymal variant. During EMT, Snail inhibits ESRP1, increasing the expression of the CD44 isoform associated with dedifferentiation and invasiveness [37]. CD44 is not, by any means, the only example of alternative splicing affecting EMT [38]. Of interest, one study found that breast cancer cells undergoing EMT exhibit a specific alternative splicing signature, with alternative isoforms of many genes correlating with alternative invasive phenotypes [39].

### Cellular junctions

As the expression of EMT-inducing genes increases, the cell surface changes dramatically. E-cadherin, a key marker of the epithelial phenotype, is a transmembrane protein responsible for anchoring neighboring cells to one another and forming adherens junctions, with its cytoplasmic component linked to the actin cytoskeleton by α- and β-catenin. Loss of this protein is required for EMT to occur, and it promotes metastasis [40]. Snail, Zeb, and Twist are well known E-cadherin repressors, which act by inducing epigenetic silencing at the E-cadherin promoter in the form of hypermethylation and histone deacetylation [41]. Expression of N-cadherin and vimentin, two proteins considered to be markers of a mesenchymal phenotype and crucial for cellular migration, are increased during this time as well. Post-translational control of E-cadherin expression at the cell surface can be acquired through O-glycosylation of the protein, which inhibits its transportation to the plasma membrane [42]. Once at the plasma membrane, E-cadherin can also be inactivated by proteolytic cleavage or destabilized by phosphorylation of β-catenin [43]. The loss of E-cadherin is an integral step in EMT and a key feature of metastatic cells. Without the tight adherens junctions keeping tissues together, individual cells are free to migrate, which is crucial for cancer metastasis.

### Receptors

Certain integrins, together with FAK signaling, play a large role in promoting migration and metastasis in cells undergoing EMT. FAK is an important tyrosine kinase, known to phosphorylate β-catenin. Once β-catenin is phosphorylated, it detaches from the E-cadherin complex and localizes to the nucleus where it promotes transcription of genes related to proliferation, migration, and invasion [43]. Wnt signaling is involved in proliferation. When Wnt is not present, β-catenin binds conductin, GSK-3β, and APC in place of E-cadherin. β-catenin’s N-terminal domain is then phosphorylated by GSK-3β, leading to degradation of β-catenin through the ubiquitin proteasome pathway. Wnt activation inhibits GSK-3β and stabilizes β-catenin, leading to its nuclear localization and increased expression of oncogenes such as c-myc and Cyclin D1. Integrin β_1_ was shown to mediate expression of FAK in lung cancer cells, with proliferation following metastasis dependent on β_1_ and FAK expression levels [44]. A different study demonstrated that nuclear localization and accumulation of Twist, along with the expression of its target gene N-cadherin, is mediated by and dependent on β_1_ integrin signaling [45]. EGFR has been found to directly interact with β-catenin as well, causing phosphorylation of β-catenin and loss of junctions. In metastasis, EGFR can induce dephosphorylation and subsequent inactivation of FAK. After metastasis, FAK is re-activated by integrin signaling during re-adhesion, showing a dynamic regulation of FAK in the processes of EMT and metastasis. Other integrins have also been linked to the induction of metastasis. For example, Snail, a known inhibitor of E-cadherin expression, also promotes expression of the α_v_β_3_ integrin, which is associated with a pro-invasive phenotype and activation of TGF-β [46,47].

### MMPs

A variety of secreted factors are also important in the maintenance of EMT and in the promotion of metastasis [48]. MMPs are capable of cleaving cell-surface proteins as well as degrading components of the extracellular matrix, allowing migratory cells to invade neighboring tissues and break through the basement membrane [49]. E-cadherin is an important substrate of MMPs, as its cleavage not only helps separate tissues into individual cells but also induces signaling supportive of EMT. Cleavage of the E-cadherin ectodomain has been shown to create a fragment, sE-cad, capable of inducing EMT, invasion, and proliferation in a paracrine manner via EGFR signaling [50,51]. Secreted cytokines have been shown to promote invasive phenotypes. For example, one study showed that ectopic expression of IL-6 was associated with E-cadherin repression and increased expression of Snail, Twist, N-cadherin, and vimentin. These findings perhaps explain the link between increased IL-6 concentrations and poor survival rates amongst breast cancer patients [52]. IL-18 has also been suggested as a marker of metastatic breast cancer and has been shown to activate MMPs while inducing secretion of other cytokines [53]. Interestingly, one study showed that IL-18 together with MMP9, was capable of inducing cardiac smooth muscle cell migration through NF-kB signaling. It is possible that in the setting of cancer, this interleukin could promote EMT and metastasis through a similar pathway [54,55].

## Clinical implications of EMT

In Table 1, the various tumor types which have thus far been most strongly correlated with EMT are presented with a brief review of known EMT markers. Cancer types are ordered by the estimated percentage of diagnosed patients who have survived 5-year following cancer metastasis. While this table is by no means exhaustive, it helps highlight several interesting trends. For example, the Snail, Twist, Zeb, and E-cadherin axis as described above, has thus far been correlated with nearly every clinically significant tumor type. Furthermore, the striking commonalities between these distinct tumors reveal the profound clinical importance of EMT as a shared, ubiquitous mechanism that promotes metastasis. Fittingly, the field of oncology has seen a recent explosion of EMT-related research for both prognostication and treatment of metastatic cancers, and to date, numerous classical EMT markers have been significantly correlated with metastasis. Moreover, recent works suggest that assessing classical markers of EMT may help clinicians predict resistance to chemotherapy, and thus poor prognosis [56].

**Table 1:** **Major tumor types organized by virulence, clinical significance, and epigenetic markers**

**Cancer type**	**Survival 5-years after cancer has metastasized [** 61 **]**	**Survival 5-years after diagnosis [** 61 **]**	**EMT Markers**	**References**
**Pancreas**	2.3%	6.7%	Snail, Twist, Zeb1, Zeb2, E-cadherin, β-catenin Brachyury, HDAC1,2,3, miR-34, miR-200,	[62-65]
**Liver**	2.8%	16.6%	Snail, Twist, Zeb1, Zeb2, TGF-β, EZH2, HDAC1,2,3, miR-101, STAT3, SUZ12,	[62,66,67]
**Lung**	4.0%	16.8%	Snail, Zeb1, Zeb2, E-cadherin, vimentin, α-catenin, EZH2, BMI1, Brachyury, Claudin-1, Cytokeratins, G9a, HDAC1,2,3, LSD1, miR-34, miR-101, miR-205, Periostin, Slug, SUZ12, TTF-1, versican, N-cadherin	[62,63,66,68-71]
**Bladder**	5.5%	77.4%	Twist, Zeb1, Zeb2, N-cadherin, EZH2, Fibronectin, LSD1, miRs-1/133a/218, miR-19a, miRs-30a-3p/133a/199a, miR-34, miR-99a/100, miR-101, miR-125b, miR-129, miR-145/133a, miR-200, miR-205, miR-221, N-	[62,66,72]
**Renal**	12.1%	72.4%	TGF-β, BMP-7, Claudin-1, HDAC1,2,3, hepatocyte growth factor, Klf8, miR-23b, miR-29b, miR-34, miRs-141/200, miR-205, miR-438-3p,	[62,66,72-74]
**Colorectal**	12.9%	64.7%	Snail, Twist, vimentin, Zeb1, Zeb2, β-catenin, Brachyury, CD44, E-cadherin, EZH2^,^ FGFR4, Fibronectin, HDAC1,2,3, LSD1, miR-34, p16^INK4a^, SIRT1, Slug, SUZ12, SUV39H1,	[62,63,66,72]
**Cervical**	16.1%	67.9%	Snail, Twist, E-cadherin, vimentin, β-catenin, EGFR,	[63,66,75,76]
**Skin melanoma**	16.1%	91.3%	TGF-β, MITF, N-cadherin, miR-205	[62,77,78]
**Ovarian**	27.4%	44.6%	Snail, Twist, Zeb1, Zeb2, E-cadherin, CCR7, Claudin-1, Fibronectin, Klf8, miR-9, miR-34, miR-200, N-cadherin, Occludin, PTEN, Slug, STK11,	[62,63,66,72,79-82]
**Breast**	25.0%	89.2%	Snail, Zeb1, Zeb2, vimentin, β-catenin, E-cadherin, BMI1, Brachyury, Claudin, EZH2, HDAC1,2,3, Klf8, LSD1, miR-9 (2); miR-10b, miR-34, Slug, SUZ12, Twist, versican,	[62,63,66,68,69,83,84]
**Prostate**	28.0%	98.9%	Twist, Zeb1, N-cadherin, APC, Cyclin D2, collagen, decorin, E47, E-cadherin, ER, EZH2, Fibronectin, GSTP1, HDAC1,2,3, Let-7a, LSD1, miR-1, miR-7, miR-15a-16 cluster, miR20a, miR-21, miR-24, miR-32, miR-34a, miR-34c, miR-101, miR-106b, miR-107, miR125b, miR-143, miR-145, miR-146a, miR-148a, miR-205, miR-221, miR-222, miR-331-3P, miR-449a, miR-521, miR-1296, Notch-1, RAR-β2, RASSF1A, versican,	[62,63,72,85-88]
**Brain/nervous system**	35.6%	33.4%	miR-9, Klf8	[62]

Another exciting area of research is the use of EMT markers in the analysis of circulating tumor cells (CTC). Diagnostically, CTC has been a mainstay of clinical practice in assessment of metastasis and prognosis. The presence of CTC in a patient’s blood can be measured using the AdnaTest, a PCR assay for markers of EMT such as Twist, Akt, and Pi3k. The test employs a method for enriching the CTCs in a blood sample using antibodies conjugated to magnetic beads. Once the tumor cells have been pulled down, the mRNA can be isolated and expression of EMT markers determined. The test is reported to be sensitive enough to detect two CTCs in a 5 mL sample of blood [57]. Recent works have indicated that consideration of CTC EMT status is critical to achieve a more accurate prognosis. In studies of metastatic breast cancer, CTC were found to express known EMT regulators, including TGF-β pathway components and the FOXC1 transcription factor. These data support a role for EMT in the blood-borne dissemination of human breast cancer. Classical markers of EMT, Twist, and vimentin, have been identified in breast cancer patients and specifically show elevated expression in patients with metastatic cancer relative to patients with early-stage cancer, supporting the hypothesis that EMT controls the metastatic potential of CTCs [58]*.* Importantly, other work suggests that EMT-CTCs may be more likely to evade classical CTC detection by the AdnaTest as a result of down-regulation of EpCAM. As suggested by Gorges et al., this may explain why patients with late metastatic cancers may report low CTC numbers, suggesting the urgent need for a better understanding of EMT-CTC in prognosis [59,60].

### Pancreatic cancer

Pancreatic cancer generally has a poor prognosis, in part because symptoms often do not appear until the cancer is too advanced for surgical treatment. Pancreatic exocrine tumors have an average 5 year survival of up to 14%. Neuroendocrine tumors have a 61% 5-year survival rate if detected at Stage 1, but these tumors are rarely detected at this phase [2,89]. Thus, early detection and inhibition of metastasis remain among the greatest challenges in the treatment of these tumors. Several genes related to EMT have been considered with respect to these clinical challenges. In one *in vitro* study, Hh inhibition with cyclopamine resulted in down-regulation of Snail and up-regulation of E-cadherin, as well as a striking reduction of invasive capacity. Combining gemcitabine and cyclopamine completely abrogated metastasis while also significantly reducing the size of “primary” tumors. These findings suggest that inhibition of the Hh pathway is a valid therapeutic strategy for pancreatic cancer that particularly targets metastasis [64,65]. Similarly, Resveratrol, which inhibits pluripotency-maintaining factors such as Kras (G12D), and EMT have been indicated in the management of pancreatic cancer [90,91].

### Hepatocellular carcinoma

Hepatocellular carcinoma (HCC), which is among the most deadly forms of cancers worldwide, is the most common primary liver cancer and is the fastest growing cause of cancer death in men in the United States [92]. The dominant risk factors are chronic Hepatitis B or Hepatitis C infection. In addition, cirrhosis can have an effect on the tumor microenvironment as well as on tumorigenesis. Cirrhosis can lead to the activation of stellate cells, which increase production of extracellular matrix proteins, cytokines, and growth factors, many of which can alter hepatocyte proliferation and promote tumorigenesis [93,94]. HCC tends to have a poor prognosis due to late diagnoses and a lack of effective treatment options. While EGFR-targeted therapies have been successful in some types of cancers, erlotinib and cetuximab have not been very effective in clinical HCC trials, particularly in the treatment of mesenchymal HCC cells. In the case of hepatic carcinomas, Sorafenib, which inhibits STAT3 and phosphorylates TGF-β which are both up-regulated in EMT, is also being studied as a potential therapeutic agent [67].

### Squamous cell carcinoma

Vimentin positive tumor cells have been detected among squamous cell carcinomas; although, high epithelial vimentin has not been correlated with tumor grade. Squamous cell carcinomas tend to have periostin rich stroma. Periostin is usually localized to the periphery of stromal cells surrounding carcinoma cells. Expression of versican and periostin were frequently accentuated toward the pseudo-basement membrane of the extracellular matrix around these carcinomas, and high stromal vimentin is associated with higher grade [71]. Since EMT plays a large role in the development and spread of lung cancer, numerous drugs that specifically target EMT are being developed or are in use in the treatment of lung cancer. For example, Sorafenib has been show to increase HAT expression in adenocarcinoma, therefore positively influencing the epigenetic profile of the cancer cells [95]. Furthermore, an immunotherapeutic approach to target a major driver of EMT, the T-box transcription factor T, also known as brachyury, is currently in Phase I clinical trial as a potential new therapy for patients with advanced lung cancer carcinomas [96,97].

### Pulmonary adenocarcinoma

Adenocarcinoma is a type of cancerous tumor that forms from glandular structures [98]. Stromal periostin protein is associated with versican collagen, and tumor cell epithelial periostin is associated with both versican and vimentin. Each of these associations suggests that cancer cells have undergone EMT and become more metastatic, but surprisingly, this study did not find a correlation between vimentin up-regulation and morphological trans-differentiation. However, the authors observed that the up-regulation of stromal vimentin, periostin, and versican is associated with higher cancer grades. As vimentin is the constituent of the cytoskeleton network, it is possible that stromal populations go through certain changes during the induction of EMT. Similar results were found in breast carcinoma [68,69].

### Urothelial carcinoma

Urothelial carcinoma makes up the majority of bladder cancers and has a high likelihood of returning after treatment. The most common treatment is surgery if the carcinoma is detected in an early stage. Urothelial cancers are further classified as either superficial or muscle invasive.

### Renal cancer

Via blood filtration, the kidneys are exposed to a disproportionately high concentration of toxins. Thus, perhaps it is not surprising that renal cancer is one of the 10 most common cancers. EMT has also been observed in mature epithelial tubular cells and has been linked to the pathogenesis of renal interstitial fibrosis. Furthermore, in mouse models it has been demonstrated that the selective blockade of EMT-associated TGF-β, hepatocyte growth factor, and BMP-7 expression reduces fibrotic lesions after obstructive injury [74].

### Colorectal cancer

Colorectal cancers tend to start as a small growth in the inner lining of the colon known as a polyp, ultimately giving rise to adenocarcinomas. Colorectal cancer is one of the most common cancers, and yet it is not among the most lethal cancers as early clinical detection via routine screenings has dramatically improved overall mortality [99]. Still, careful study of EMT markers has revealed additional clinically relevant information. A clear link has been established between CD44, enhancement of EMT, and colon cancer invasion [100]. Furthermore, FGFR4 has also been shown to play a crucial role in tumorigenesis, invasion, and survival in colorectal cancer, and its specific targeting marks a new avenue of colorectal cancer therapy [101]. Vimentin is highly expressed in the stroma of colorectal cancer cells compared to healthy cells, but interestingly, not in the cancer cells themselves. Higher levels of stromal vimentin have been correlated with poor prognosis of colorectal cancer. Specifically, since vimentin is expressed in mesenchymal cells and not epithelial cells, it indicates that EMT has taken place [102].

### Cervical cancer

Perhaps the most significant recent breakthroughs with respect to cervical cancer have come from the understanding that human papilloma virus (HPV) silences tumor suppressor genes through production of proteins E6 and E7. However, as worldwide immunization campaigns evolve, cervical cancer persists as a clinical challenge, and stage IV cervical cancer is still generally considered untreatable, though chemotherapy is recommended which uses platinum drugs [103]. Several EMT genes have recently been explored as potential biomarkers or targets of drug treatment in cervical cancer. For example, FTS silencing was found to reduce EMT and cell migration by EGF treatment [104]. Importantly, Twist2 has been identified as the key Twist isoform coupling aberrant signals from EMT to senescence, with significant implications on its potential utility as a biomarker of cervical cancer prognosis [75,76].

### Melanoma

TGF-β and EMT regulation markers such as MITF have been shown to play a critical role in melanoma progression. Furthermore, up-regulation of N-cadherin has been correlated with an increase in cell migration and invasion. Recent works have demonstrated the causal role of TGF-β-induced EMT-like changes on downstream activation of PI3K in human melanoma cells, which may ultimately yield new therapeutic options for these highly aggressive cancers [77]. Another significant recent insight has been that the EMT-like switch in phenotype is associated with a concomitant change in the expression of multiple tumor antigens, ultimately allowing cells to evade T-cell killing. This may have important implications for future immune therapies such as cancer vaccination, and careful selection of target antigens may help circumvent the problem of T-cell evasion by metastatic melanoma cells [78].

### Ovarian cancer

Mutations in the BRCA1 and BRCA2 genes may contribute to development of ovarian cancer. PTEN and STK11 (a tumor suppressor protein related to EMT) may also be risk factors. Furthermore, CCR7, which can be induced in response to hypoxia and is often constitutively expressed in epithelial ovarian cancer cells, has been shown to participate in EMT development, leading to cell migration and invasion. This suggests that CCR7 may be an effective target for limiting cell invasion in certain ovarian cancers [80]. Other recent work has linked hTERT to Slug expression in norepinephrine-induced ovarian cancer EMT and metastasis. This suggests that these genes may serve as novel biomarkers and potential therapeutic targets for ovarian cancer [81,82].

### Breast cancer

CTCs that have undergone EMT have been found in patients with HER2 (+) metastatic breast cancer. CD326 (−) and CD45 (−) cells show an enrichment of circulating stem cells (CSCs), and have been shown to be correlated with classical markers of EMT such as Snail1 and Zeb1 [84]. Therefore, assessing EMT-CTCs and CSCs in HER2 (+) breast cancer patients could be of great prognostic value [84]. Additionally, high levels of CD44 and low levels of CD24 have been linked to chemotherapy resistance and cancer relapse in metastatic breast cancer*.* Clinically, Lapatinib in combination with conventional therapy, was demonstrated as a possible therapeutic strategy for eliminating these cells to decrease recurrence and improve long-term survival [105].

### Prostate cancer

During prostate cancer progression, as the cells undergo EMT, the stroma undergoes structural rearrangement in order to accommodate the tumor cell. Tumor cells can evade apoptosis by changing their relationship to the ECM. One marker of a reactive stroma is the presence of myofibroblasts, which is a cellular intermediate between fibroblasts and smooth muscle cells [106]. These cells secrete fibronectin, collagen, and proteoglycans such as versican and decorin [107-111]. The reactive stroma is not only responsible for assisting in EMT but also contributes to tumor vascularization [112]. Aberrant glycosylation also impacts such EMT and cell adhesion [113]. Several patterns of gene silencing have been documented in advancing prostate cancer. Genes such as APC, RASSF1A, CCND2, and RAR-β2 are silenced even in less virulent (low Gleason score) tumors, and loss of E-cadherin, GSTP1, and ER tend to be silenced in more aggressive tumors [85-88]. Approximately half of prostate cancers carry TMPRSS2-ERG translocations; however, the clinical impact of this genomic alteration remains unclear. Recent studies have suggested that ILK is a therapeutically targetable mediator of ERG-induced EMT and transformation in prostate cancer [114].

### Glioblastoma

Arising from astrocytes, glioblastoma is the most common primary and most aggressive CNS tumor subtype. A particular challenge to treat, the tumors are generally very heterogeneous, and thus, some cells may respond to treatment while others may not. Glioblastomas are highly malignant in part because they reproduce quickly and have access to many blood vessels, but rarely spread to distant locations in the body. In glioblastoma multiform, EMT has been shown to cooperate with MMP activity, allowing cells to gain access to lymph vessels. Preliminary data suggest this new EMT-associated drug target in combination with stereotactic radiosurgery may provide potential targets for future treatment [115].

### Smoking

Though beyond the scope of this paper, the role of smoking in the pathogenesis of EMT is also of high clinical significance. Recent works have established direct connections between cigarette smoke and acute inflammatory mechanisms such as NF-kB and EMT [116]. Although the bulk of the evidence for this relationship has been considered with respect to lung cancers, it is likely that these mechanisms will be more explicitly implicated for other tumor types as well. Thus, epidemiologically, smoking cessation may ultimately prove among the most important clinical interventions relevant to EMT.

## Epigenetics, EMT-MET, and Metastasis

As described in the introduction, embryogenesis is a process that involves growth and differentiation and is regulated primarily by epigenetic events. Opportunistic cancer cells and cancer progenitor cells hijack this process to their advantage to go through EMT and possibly the opposite process of MET for successful metastasis. The previous paradigm proposed by many researchers that metastatic processes involve the accumulation of mutations and genetic changes does not explain the reversible phenomena of EMT and MET, as mutations and genetic changes are irreversible. For this reason, Sarkar et al. previously proposed that the initiation of carcinogenesis and EMT/MET processes should be regulated by epigenetic mechanisms which are, by default, reversible [2,17,18,23,117,118]. While mutations and other genetic alterations can speed up cancer cell growth at a certain degrees of metastasis, the amount of EMT defining a particular degree of differentiation should be controlled by epigenetic changes [17]. These epigenetic changes involve histone modifications, DNA methylation, and changes in the expression of miRNA. Tam and Weinberg also recently proposed that epigenetic changes are involved in the stepwise progression of EMT [119], but they do not describe the changes necessary at different times to produce metastatic cancers of different grades. As explained by Sarkar et al., these epigenetic changes are grade-specific and variable because they occur at different times, when growth slows down and differentiation speeds up. Once the differentiation for the more metastatic form is achieved, growth speeds up and new mutations incurred at that time may help in this process of rapid growth. The reverse process takes place during MET. Thus, epigenetic changes that promote and enable both EMT and MET are dynamic and variable, not static. Overall, the involvement of epigenetic changes in cancer is well studied, and the involvement of epigenetic changes in cancer initiation are discussed elsewhere [17,18,23,117,118]. Therefore, in this review we instead attempt to connect the significance of epigenetic changes related to EMT and MET.

As discussed in the signaling section, TGF-β, cadherin, and integrins play significant roles in EMT. Interestingly, E-cadherin and integrin α4 are silenced by methylation during EMT. TGF-β receptors are functional during EMT as they drive the differentiation process, but these receptors are silenced by methylation in terminal grade cancer. This suggests that differentiation is not required at the terminal grade of metastatic cancer, and therefore, these receptors are silenced [120]. The epigenetic regulation of TFG-β1 during EMT is supported by a recent study which demonstrated that HDAC inhibition suppresses EMT induced by TGF-β1 in human renal epithelial cells [121]. Sarkar et al. has previously shown that in addition to increasing acetylation levels in histones, HDAC inhibitors also demethylate CpG residues by down-regulating DNMT1 [122-124].

**Figure 1:**
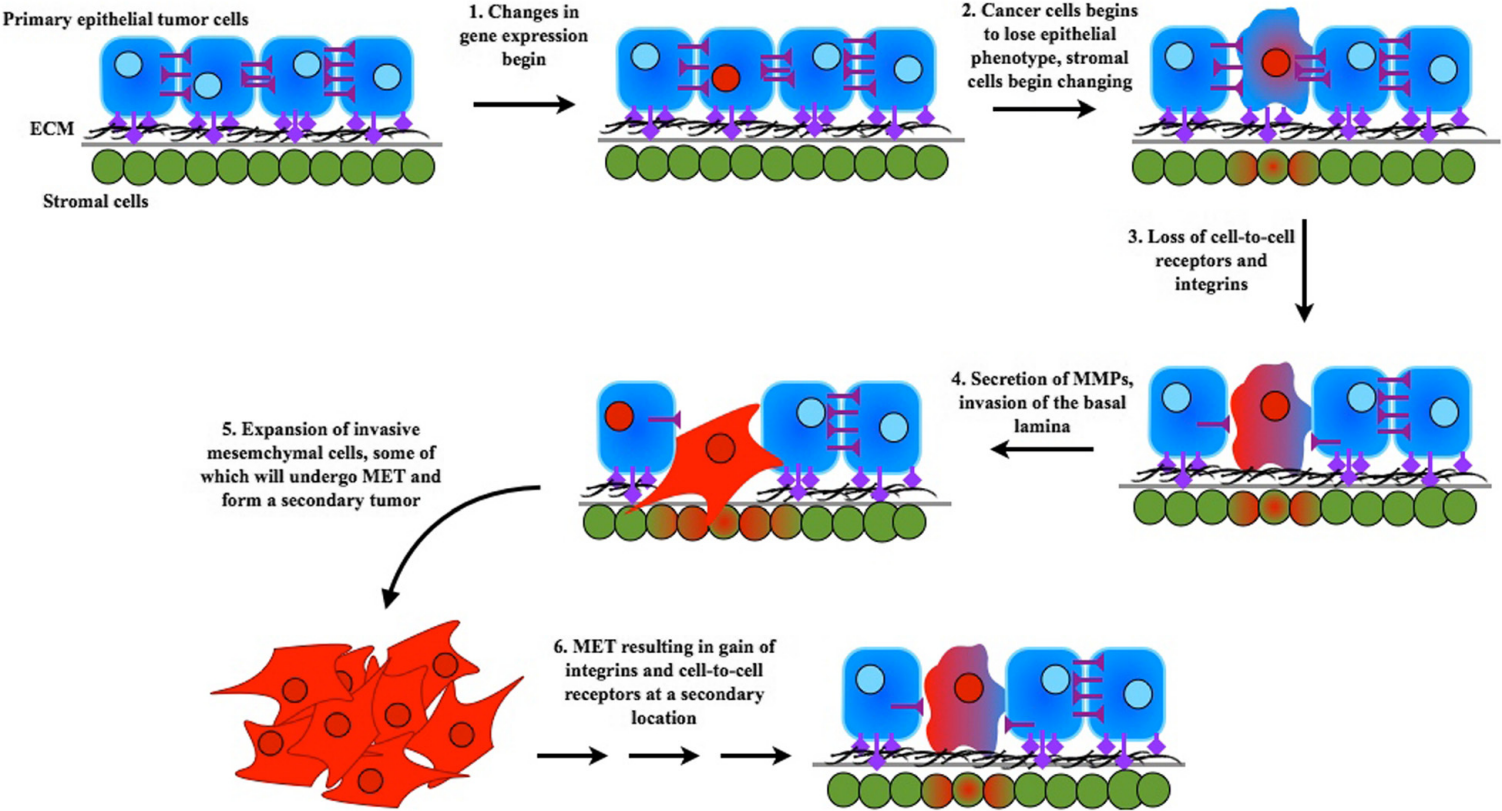
**Changes that occur as a tumor cell undergoes EMT and then metastasizes at a secondary location.** Epithelial tumor cells are shown in blue, and stromal cells are shown in green. As a tumor cell undergoes EMT, it begins to lose its epithelial phenotype as shown after step 2. Loss of cell-to-cell attachment receptors and integrins (shown in purple) also occurs and continues to step 3 and beyond. In addition, stromal cells near the cancer cell (which is undergoing EMT) are affected and begin undergoing changes (shown as a progression from green to red cells). Once a cancer cell has completely undergone EMT and travels to a new location, multiple steps (not explicitly shown) involving MET must occur for the metastatic cancer cell to anchor to the distant site and form a secondary tumor. The stromal cells at the new tumor location will also undergo change.

As cancer progenitor cells go through EMT, their morphology changes, and that requires rearrangement of the cytoskeleton (Figure 1). Vimentin is an important constituent of the cytoskeleton, whose expression goes up in many types of cancer during EMT and in the stromal cells of several cancers, such as non-small-cell lung cancer and colorectal cancer [70,102]. Additionally, methylation pattern changes have been observed in the stromal cells of metastatic breast cancer as far as 4 cm from the primary tumors [125]. These results suggest that stromal cells also go through epigenetic changes that regulate their morphology and function during EMT. Thus, cancer progenitor cells and stromal cells may communicate and exchange signaling materials, possibly through paracrine mechanisms involving cytokines [126], during EMT. While cancer progenitor cells undergo differentiation and shape changes, stromal cells close to progenitor cells may also experience morphology change and perhaps differentiation (Figure 1). It is well known that epigenetic changes occur in cancer progenitor cells, but epigenetic changes also occur in stromal cells [125]. However, this model does not suggest that the degree of differentiation for all grades of metastatic cancer happen simultaneously or at a particular point in time. Rather, this model depicts only the gradual process of metastasis (Figure 1), which involves gradual change in epigenetic regulation. As discussed earlier, such changes are involved in forming different degrees of cancer metastasis [2,17,18,25,117,118]. Following EMT and relocation, cancer cells go through MET so as to attach to the epithelium.

Epigenetic drift, which involves age dependent changes in genomic methylation patterns, is a new area of interest that may be relevant to cancer. This phenomenon of epigenetic drift may be tissue specific or tissue independent, and it results in stem cell differentiation processes becoming less flexible with age [127]. Interestingly, cancers and precursor cancer cells from lesions of advanced metastatic tumors also demonstrate this type of epigenetic drift, indicating that cancer progenitor cells hijack these age-related normal changes in epigenetic events in order to promote EMT for metastasis.

After EMT and once the metastatic cancer cells migrate away from the original tumor, they need to anchor to distant tissues and organs for successful tumor development at the new site. This process requires a reversal of EMT, or MET, and the re-expression of molecules that will help those metastatic cancer cells transition to attach to the new tissue. Although the role of MET in cancer is a new area of study, recent findings on the reprogramming of somatic cells to iPSCs reveal that a key role of BMP signaling is the induction of MET during the initiation phase. Interestingly, the miR-205 and miR-200 family of miRNAs were involved in this induction process [128]. Down-regulation of miR-34c has been shown to cause EMT in breast cancer initiating cells [129]. Another recent study has shown that silencing of TET mediated demethylation of anti-metastatic miR-200 promotes metastasis in a transgenic mouse breast cancer model. This process is regulated by miR-22 [130,131]. Interestingly, it has been observed that some of miRNA expression is regulated by methylation [132,133]. Other noncoding RNAs, such as long non-coding RNAs (lncRNA), have also been shown to be under epigenetic control and to have a role in regulating EMT. For example, lncRNA H19, which suppresses E-cadherin [134], is hypomethylated in bladder cancer, leading to more metastatic cancer progression [135]. The up-regulation of lncRNA MALAT1 also induces EMT in bladder cancer [136]. Additionally, several other cancers are influenced by alterations in lncRNA regulation, which are often induced by Twist and regulate Wnt downstream [137-139]. These results suggest that epigenetics is involved in EMT/MET processes.

After therapies and the apparent remission of the cancer, patients often relapse. One of the possible reasons for relapse is the survival of cancer progenitor cells, and drug resistant cancer cells at the site of the tumor [17,18,23,117,118]. Another issue is the presence of circulating cancer progenitor cells. This is currently a major field of study, as these cells have the potential to cause cancer relapse [140]. The presence of these cells in a patient who is in remission is an indication of possible cancer relapse. Additionally, as these cells have undergone EMT and are in a metastatic state, they need to go through MET to anchor in distant tissues and form new tumors. The issue of metastatic cancer progenitor cell colonization has previously been discussed by Chaffer and Weinberg [41,48]. Sarkar et al. suggested that this localization should involve MET [17]. As lung cancer metastasis is faster than breast and prostate cancer metastasis in a relapse scenario, it is possible that MET is faster in lung cancer.

Many clinical and research studies suggest that pre-treatment of cancer patients with epigenetic drugs reduces cancer relapse [117,118]. These studies indicate that inhibition of epigenetic processes may kill cancer progenitor cells and drug resistant cancer cells, inhibit EMT, and possibly inhibit MET in circulating cancer progenitor cells. This topic was discussed in more detail elsewhere [17].

## Conclusion

The metastasis process is different from the initiation and progression of cancer in that not all of the transformed cells become metastatic. The current paradigm states that a few of the transformed cancer cells, presumably a few of the cancer progenitor cells, go through EMT to produce a metastatic form of cancer. This is a very complex process, and as described in this review, it is regulated by diverse mechanisms. As the metastasis includes both EMT and MET, we believe that this should be a reversible phenomenon. In the cellular context, we compare this phenomenon with embryogenesis and normal development which are both regulated by epigenetic changes, such as histone modifications and DNA methylation and demethylation. One recent study notes that DNMT1 efficiency is higher in cancer cells as compared to that in normal cells [141]. This finding opens a new area of study to determine how the generation of methylated regions by highly efficient DNMT1 proteins, which regulate enhancer and transcription factor interactions and gene expression, influences carcinogenesis. Additionally, recent computational biology studies have used enhancer analysis to combine genetic and epigenetic events in the prediction of gene regulation and expression. They seem to be tissue specific [142]. In disease conditions including in cancer, the enhancer pattern alterations are more at par with epigenetic changes rather than mutational and other changes. The insulated region created by CTCF does not allow gene expression and most developmentally regulated genes, and stem cell pluripotency genes are regulated this way [143]. The insulated region alters during changes in methylation levels in cancer cells [144]. This approach will be valuable to test the hypotheses we have provided in this review and in previous publications about cancer progenitor cell formation, cancer progression, EMT/MET, and metastasis [17,18,23,117,118]. Understanding these complex processes will help in developing improved chemotherapies that could be used to inhibit metastasis. Many of the anticancer drugs that inhibit growth and induce tumor cell death are not capable of inhibiting metastasis. Some of the drugs that show promise in mouse models fail to stop tumor growth and metastasis in humans. While almost all signaling and genetic events are similar in mice and xenograft tumor models, the role of the stroma as described in this review may be different in mice and humans and produce different outcomes. Interestingly, pretreatment with epigenetic drugs in a combination therapy does reduce the relapse of cancer. Elucidation of the exact steps of EMT will help in the development of improved antimetastatic therapies that are useful against circulating metastatic cancer cells and drug resistant cancer cells.

## Abbreviations

EMT: Epithelial-mesenchymal transition
TGF-β: Transforming growth factor beta
MET: Mesenchymal-epithelial transition
iPSCs: Induced pluripotent stem cells
Zeb: Zinc finger E-box binding homeobox
HNF-1β: Hepatocyte nuclear factor 1 homeobox B
HIF: Hypoxia-inducible factors
NF-κB: Nuclear factor kappa-light-chain-enhancer of activated B cells
IGFR: Insulin-like growth factor 1 receptor
CCN6: Cysteine-rich secreted protein
WISP3: Wnt1 inducible signaling pathway protein 3
IGF-1: Insulin-like growth factor 1
Cox-2: Cytochrome c oxidase 2
IL: Interleukin
VEGF: Vascular endothelial growth factor
ER: Estrogen receptor
ERE: Estrogen-responsive element
HRE: Hormone response element
FAK: Focal adhesion kinase
GSK-3β: Glycogen synthase kinase 3 beta
APC: Adenomatous polyposis coli
EGFR: Epidermal growth factor receptor
MMP: Matrix metalloproteinase
sE-cad: se-cadherin
CTC: Circulating tumor cells
FOXC1: Forkhead box C1
HER2: Human epidermal growth factor receptor 2
CSC: Cancer stem cells
EpCAM: Epithelial cellular adhesion molecule
HAT: Histone acetyl transferase
SIP1: Smad interacting protein 1
Hh: Hedgehog
Kras: V-Ki-ras2 Kirsten rat sarcoma viral oncogene homolog
ECM: Extracellular matrix
RASSF1A: Ras association domain-containing protein 1A
RAR-β2: Retinoid acid receptor beta 2
GSTP1: Lutathione S-transferase pi 1
FTS: Fused Toes Homolog
EGF: Epidermal growth factor
BRCA: Breast cancer
PTEN: Phosphatase and tensin homolog
STK11: Serine/threonine kinase 11
CCR7: Chemokine receptor 7
hTERT: Telomerase reverse transcriptase
HCC: Hepatocellular carcinoma
TMZ: Temozolomide
HDAC: Histone deacetylase
DNMT1: DNA methyltransferase 1
BMP: Bone morphogenic protein
TET: Ten-eleven translocation methylcytosine dioxygenase
